# Advanced materials for flexible and wearable energy storage devices

**DOI:** 10.1039/d6ra01725h

**Published:** 2026-07-02

**Authors:** Mervat Ibrahim, Hani Nasser Abdelhamid

**Affiliations:** a Zhejiang Carbon Neutral Innovation Institute, Zhejiang International Cooperation Base for Science and Technology on Carbon Emission Reduction and Monitoring, College of Materials Science and Engineering, Zhejiang University of Technology Hangzhou 310014 China; b Chemistry Department, Faculty of Science, New Valley University El-Kharja 72511 Egypt Mervatibrahim1990@sci.nvu.edu.eg; c Department of Chemistry, College of Science, Imam Mohammad Ibn Saud Islamic University (IMSIU) Riyadh Saudi Arabia hnabdelhamid@imamu.edu.sa

## Abstract

The rapid advancement of wearable electronics has intensified the need for lightweight, flexible, deformable, and self-sustaining energy-storage systems. Flexible supercapacitors (FSCs) have emerged as promising options for wearable energy-storage applications due to their high-power density, short charge–discharge times, long cycling stability, and mechanical flexibility. Recent advancements in materials engineering, additive manufacturing, and self-sustaining systems have improved the electrochemical and mechanical efficacy of FSCs. This review discusses recent advancements in improved materials, fabrication techniques, and integrated self-charging systems for next-generation wearable supercapacitors. It covers most of the reported materials, including conductive polymers, carbon nanomaterials, MXenes, metal oxides, metal–organic frameworks (MOFs), covalent organic frameworks (COFs), and hybrid nanocomposites. Advanced fabrication techniques, such as three-dimensional (3D) printing, microfluidic spinning, wet spinning, dry spinning, coating deposition, screen printing, laser writing, and hybrid UV-assisted 3D printing, are highlighted concerning their impact on electrode structure, ion transport, conductivity, and mechanical integrity. Hybrid material that integrate electrical double-layer capacitance (EDLC) with pseudocapacitive charge-storage processes are highlighted as efficient approaches to improve energy density and electrochemical performance. Furthermore, recent developments in wearable self-charging power systems that integrate triboelectric generators (TENGs) with FSCs are highlighted, illustrating the viability of harvesting biomechanical energy to enable uninterrupted, autonomous device operation.

## Abstract 2

This review summarizes recent advances in flexible supercapacitors (FSCs) for wearable and self-powered electronics, focusing on advanced electrode materials.

## Graphical abstract

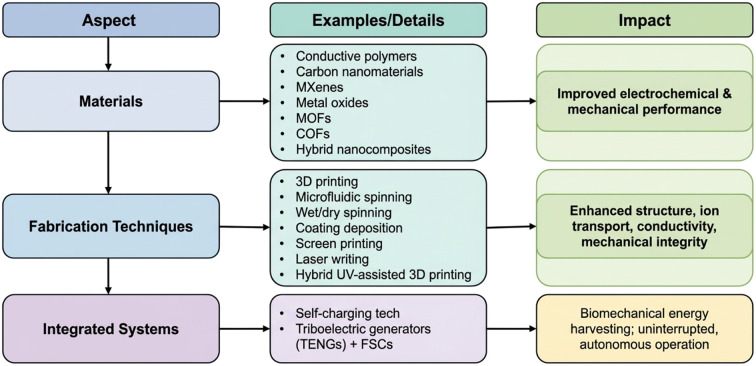

## Introduction

As the world confronts the dual challenges of fossil fuel depletion and escalating environmental degradation, the global energy landscape is undergoing a necessary transformation.^1,2^ The continued dependence on petroleum and natural gas has not only driven unprecedented industrial and technological progress but has also intensified concerns regarding energy security, environmental pollution, and greenhouse gas emissions.^3–5^ Consequently, sustainable and renewable energy technologies are increasingly recognized as essential for achieving global energy sustainability and offer long-term economic development.^6–9^ Because of these advancements, electrochemical energy storage (EES) technologies play an important role in stabilizing renewable energy systems and enabling next-generation electronic devices.^10–12^

Over the past three decades, EES technologies such as lithium-ion batteries (LIBs),^13,14^ Zn-ion hybrid supercapacitor,^15^ and supercapacitors (SCs) have experienced remarkable technological and commercial progress.^16–19^ These devices currently power a wide range of applications, including portable electronics and electric vehicles.^20^ However, conventional EES systems remain inherently rigid, bulky, and mechanically fragile, characteristics that are increasingly incompatible with the rapidly expanding field of wearable and flexible electronics.^21–24^ As modern electronic devices evolve toward lightweight, portable, and multifunctional platforms, there is a high demand for EES capable of meeting both mechanical and electrochemical requirements. Meeting these requirements remains a major challenge. Conventional batteries rely on rigid current collectors, brittle electrode materials, and liquid electrolytes, making them unsuitable for flexible configurations. To address these limitations, significant efforts have been devoted to developing flexible electrodes, solid-state or gel polymer electrolytes, with suitable structural architectures that preserve electrochemical stability under repeated mechanical deformation. Planar and fiber-based device configurations have emerged as promising strategies for integrating energy storage systems directly into textiles and wearable platforms. Fiber-shaped supercapacitors exhibited high stretchability.^25^ Beyond mechanical compliance, these architectures also enable multifunctional properties, such as stretchability, self-healing, and integration with energy-harvesting technologies.^26^

The past decade has witnessed a significant increase in wearable electronics, with the market size nearing 100 billion USD.^27^ Despite substantial progress, several critical challenges continue to hinder the widespread adoption of flexible and wearable EES,^28–32^ including batteries,^33–35^ and supercapacitors.^23,36–40^ A persistent trade-off exists between mechanical flexibility and energy density, where higher-capacity systems often exhibit reduced structural robustness. Furthermore, maintaining high-rate capability, long-term cycling stability, and environmental durability under varying humidity, temperature, and mechanical stress conditions remains difficult. Safety concerns, including electrolyte leakage, overheating, and thermal runaway, cause significant barriers to commercialization and consumer acceptance. Given these challenges, a comprehensive evaluation of recent advances and remaining limitations in flexible energy storage technologies is both timely and necessary. Although numerous reviews have addressed specific aspects such as material design, electrochemical mechanisms, or fabrication strategies, relatively few studies have provided an integrated perspective linking material innovation, device architecture, and practical system-level implementation.^39^ Self-powder devices enable several important applications such as transistors,^41^ sensors,^42–44^ smart textiles,^45^ self-healing electronics,^46^ photodetectors.^47^ Three-dimensional (3D) structures enabled high electrochemical performance.^48^

**Fig. 1:**
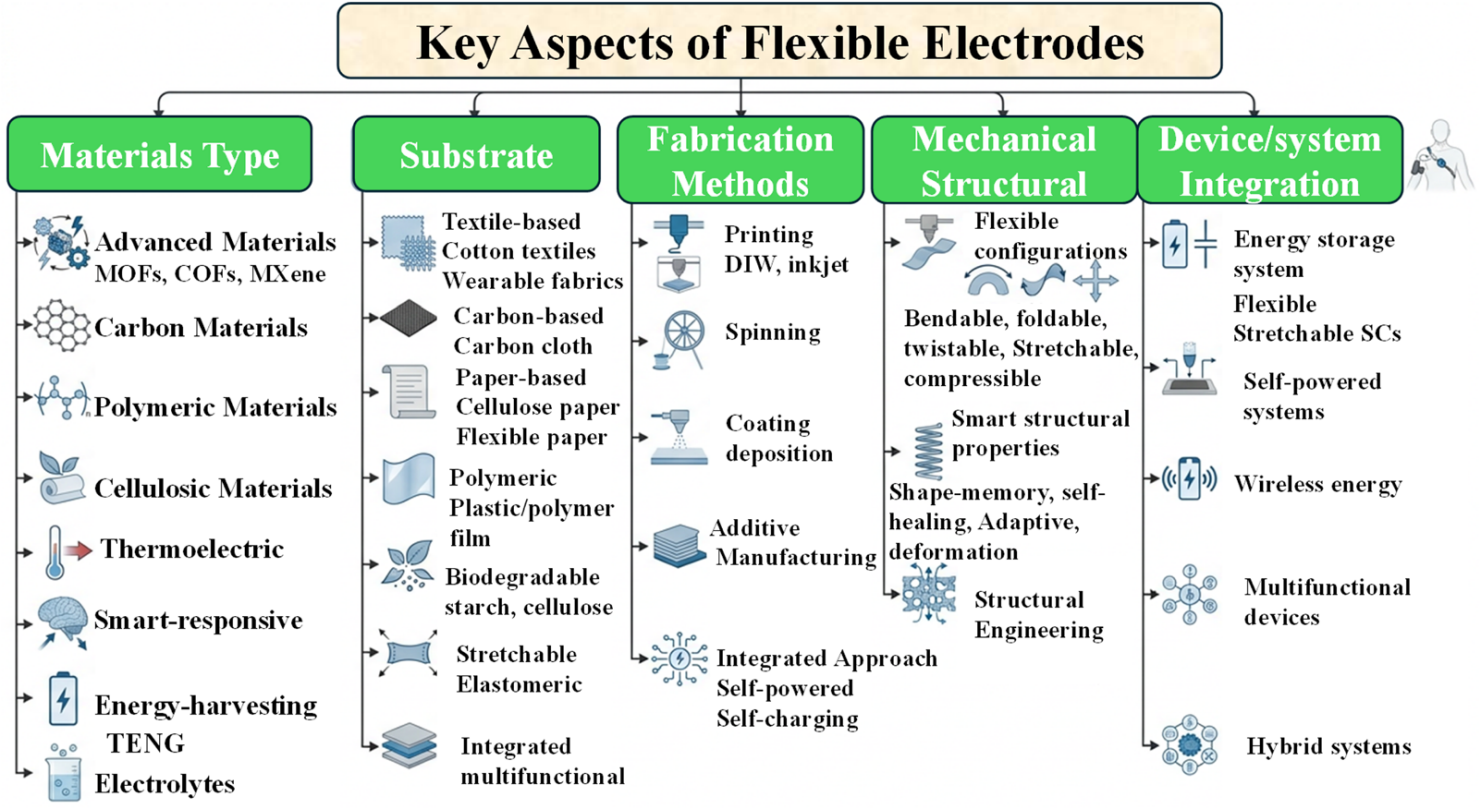
Overview of the topic summarized in this review.

This review aims to connect laboratory-scale advancements with the practical application of flexible and wearable energy-storage systems by offering a thorough overview of recent progress in materials, device architectures, fabrication technologies, and integrated self-powered systems (Fig. 1). Significant focus is directed towards the synergistic interplay among advanced functional materials, structural engineering, and system-integration strategies, which collectively influence the electrochemical performance, mechanical durability, flexibility, and practical applicability of wearable energy-storage technologies. The review initially examines the essential design ideas and material specifications required to attain superior electrochemical performance while ensuring outstanding mechanical flexibility, stretchability, and deformability (Fig. 1). Multiple configurations of flexible and stretchable supercapacitors that can withstand bending, folding, twisting, compression, and stretching are analyzed. Furthermore, innovative smart-responsive supercapacitors that exhibit intelligent features such as self-healing, shape-memory, strain responsiveness, and adaptive mechanical properties are emphasized for next-generation wearable electronics (Fig. 1). A complete assessment of advanced electrode materials and architectures is presented, encompassing flexible thermoelectric electrodes, carbon-cloth-based electrodes, polymeric electrodes, and electrodes sourced from sustainable cellulosic materials, including cellulose, paper, starch, cotton, and cotton textiles. Special emphasis is placed on biodegradable and environmentally sustainable materials that can simultaneously provide mechanical strength, conductivity, flexibility, and cost-effective processing. The review examines the advancement of self-healing and shape-memory supercapacitors to enhance long-term durability and operational reliability under repetitive mechanical deformation. Recent advancements in fabrication technologies are critically examined, encompassing screen printing, direct ink writing, inkjet printing, wet spinning, dry spinning, microfluidic spinning, laser-assisted processing, coating deposition, and 3D-printing techniques to produce flexible supercapacitor electrodes and integrated devices. These manufacturing methods enable controlled regulation of electrode shape, porosity, conductivity, active material loading, and ion-transport pathways (Fig. 1). The review emphasizes the multifunctionality of 3D-printed supercapacitors, which can be integrated into intelligent systems that demonstrate electrochromic, thermoresponsive, sensing, and self-powered features. Moreover, wearable wireless-charging power systems and integrated self-charging platforms that combine energy harvesting with energy storage are examined as alternatives for attaining energy-autonomous wearable electronics. The combination of triboelectric nanogenerators, thermoelectric systems, and flexible supercapacitors shows significant potential as a continuous, sustainable power source for wearable applications. The review concludes by identifying the primary challenges and prospective research avenues for flexible and wearable energy-storage systems, encompassing scalability, production costs, safety, electrolyte stability, mechanical durability, washability, biocompatibility, and environmental sustainability. The advancement of multifunctional hybrid materials, scalable additive manufacturing methods, self-healing electrolytes, and highly integrated energy-harvesting and storage systems is expected to expedite the commercialization of next-generation wearable and sustainable electronic devices.

## Supercapacitors (SCs)

The rapid growth of wearable electronics has generated increasing demand for lightweight, flexible, and mechanically robust energy storage systems.^49,50^ Although Li-ion batteries dominate the current EES market, their limited flexibility, safety concerns, and poor mechanical stability under repeated deformation restrict their application in flexible electronics. These limitations have stimulated extensive research into alternative energy storage technologies capable of maintaining high electrochemical performance under mechanical stress.

Among the available technologies, SCs have emerged as highly promising candidates for flexible energy storage applications due to their high-power density, rapid charge/discharge, long cycling life, and intrinsic operational safety.^51^ SCs store energy either through electrostatic ion adsorption in electrical double-layer capacitors (EDLCs) or through fast surface redox reactions in pseudocapacitors,^52^ offering rapid energy delivery and exceptional cycling stability over millions of cycles, making SCs particularly attractive for wearable and flexible electronic systems.^23,34^

**Fig. 2:**
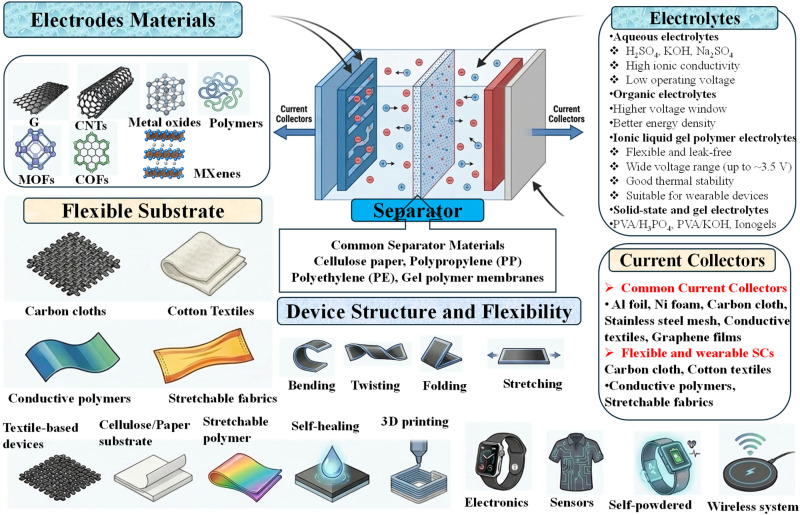
Components and applications for supercapacitors.

A supercapacitor primarily has four components: electrodes, electrolytes, separators, and current collectors (Fig. 2). The electrodes serve as the primary energy-storage components and are often made of carbon-based materials, *e.g.* activated carbon (AC), graphene, carbon nanotubes (CNTs), and reduced graphene oxide (rGO), owing to their high surface area, conductivity, and cycle stability. Conductive polymers, *e.g.*, polyaniline (PANI), and polypyrrole (PPy), offer enhanced pseudocapacitance and flexibility, whilst metal oxides and chalcogenides deliver substantial theoretical capacitance *via* redox processes. Advanced materials such as MXenes and hybrid nanocomposites significantly enhance conductivity, ion transport, and mechanical resilience. Hybrid materials are significant important because they integrate EDLC and pseudocapacitive mechanisms to enhance energy storage. Examples comprise carbon–metal oxide composites, carbon–conductive polymer systems, MXene-based hybrids, and metal oxide nanocomposites. These materials enhance conductivity, ion transport, electrochemical activity, and mechanical strength.

Recent advancements in flexible and wearable supercapacitors have concentrated on creating lightweight, pliable, extensible, and self-repairing devices for next-generation electronics (Fig. 2). Flexible electrodes have been constructed with carbon cloth, conductive polymers, metal oxides, cellulose, paper, starch, cotton textiles, and various sustainable materials. These devices can endure bending, folding, twisting, compression, and stretching while preserving electrochemical performance (Fig. 2). Smart-responsive supercapacitors exhibiting shape-memory characteristics, strain responsiveness, and self-healing capabilities have been developed for advanced wearable applications.

Advanced fabrication processes have expedited the advancement of flexible supercapacitors. Techniques such as direct ink writing (DIW), screen printing, wet spinning, dry spinning, microfluidic spinning, coating deposition, laser-assisted processing, and 3D printing enable precise control of electrode structure, porosity, conductivity, and active material incorporation. DIW and additive manufacturing are prevalent because to its capability to produce intricate 3D structures with substantial mass loading and adjustable shapes. Multifunctional 3D-printed supercapacitors have also exhibited electrochromic, thermal-responsive, sensing, and self-powered capabilities.

Notwithstanding significant advancements, numerous challenges continue to impede the commercialization of wearable supercapacitors. These include comparatively low energy density compared to batteries, electrode deterioration under repeated deformation, electrolyte instability, washability issues, limited scalability, high production costs, and challenges in integrating energy harvesting with energy storage. Subsequent research will focus on multifunctional hybrid materials, self-healing electrolytes, scalable additive manufacturing techniques, biodegradable substrates, and integrated self-powered systems that combine energy-harvesting technologies, including triboelectric and thermoelectric generators, with flexible supercapacitors.

Electrolytes are important components of supercapacitors.^53,54^ Electrolytes enable ion transfer between electrodes and may be aqueous, organic, ionic-liquid-based, or gel-polymer electrolytes (Fig. 2). Ionic liquid gel polymer electrolytes (IL-GPEs) are highly appealing for flexible and wearable supercapacitors, offering high ionic conductivity, wide operating voltage ranges, thermal stability, flexibility, and self-healing properties. Separators are permeable insulating membranes that inhibit electrical short circuits while facilitating ionic flow, whereas current collectors, including aluminum (Al) foil, nickel (Ni) foam, carbon cloth (CC), conductive textiles, and graphene sheets, convey electrons to the external circuit (Fig. 2). GPEs consist of a conductive liquid phase encapsulated within a polymer matrix.^55^ IL-GPEs or ionogels, have garnered considerable interest for FSC applications. IL-GPEs comprise ionic liquids encapsulated within a polymer matrix, merging the solid-like mechanical stability of polymers with the liquid-like ionic conductivity of ionic liquids. IL-GPEs demonstrate numerous beneficial characteristics, including as high ionic conductivity, extensive electrochemical operating windows of up to 3.5 V, remarkable thermal stability, and wide operational temperature ranges. Moreover, their tunable chemical properties and self-repairing abilities make them exceptionally promising for advancing, versatile, and resilient FSC. Another significant benefit is that FSCs with ionogel electrolytes often eliminate the need for a separate membrane separator between the electrodes, thereby streamlining device layout and reducing manufacturing costs. Owing to their distinctive characteristics, IL-GPEs have emerged as a significant area of interest in EES.^55^ Contemporary research primarily focuses on their categorization, molecular architecture, ionic transport characteristics, and incorporation into adaptable energy storage systems. Recent advancements have shown enhanced electrochemical performance, mechanical flexibility, and device stability; nonetheless, issues of cost, large-scale production, and long-term durability persist.^56,57^

### Materials used for supercapacitors

#### Carbon materials for supercapacitors

There are several carbon materials, *e.g.*, rGO,^58^ graphene (G),^59,60^ carbon nanotubes (CNTs),^61^ graphene fibers,^62^ carbon fiber,^63,64^ and carbon nanofibers^65^ (Table 1). Carbon-based materials enabled high-performance supercapacitors.^66^ Among these components, electrode materials play the most critical role in determining capacitance, charge storage behavior, mechanical durability, and overall device efficiency. Ideal electrode materials must combine high electrical conductivity, excellent electrochemical activity, and strong resistance to repeated deformation. Carbon-based materials such as G and CNTs have attracted extensive attention owing to their high conductivity, large specific surface area, and superior electrochemical characteristics.^11,32,53^ Carbon nanomaterials enabled binder-free electrodes^67^ and freestanding electrodes.^68^ They can be prepared from cheap and abundant sources, *e.g.*, textile fabrics.^69^ They can be shaped into different forms, such as aerogels.^70^

**Table 1:** Summary for materials, methods, and electrochemical performance of different materials

Materials	Procedure	Substrate	Loading	Capacitance	Scan rate	Energy density	Power density	Retention, cycles no.	Ref.
MoS_2_/CNTs	DIW	PVDF-HFP	3.2 g MoS_2_-CNTs	723 F g^−1^	mV s^−1^	226 mWh g^−1^		95% after 10 000 cycles	72
MXene/CNTs	Self-stacking and cutting	MXene/CNTs	80 wt%	550 F g^−1^	2 mV s^−1^	7.34 Wh kg^−1^	50 W kg^−1^	99% after 5000 cycles	77
Ti_3_C_2_T_*x*_	Grooving	Carbon cloths		820 F g^−1^	1 mV s^−1^	58.74 Wh kg^−1^	1.56 W cm^−12^	99.37%, 10 000 cycles	105
BC-PANI/N-MXene	Freeze polymerization technique	PANI	4.5 wt%	5525 mF cm^−2^ (635 F g^−1^)	5 mA cm^−2^	20 Wh kg^−1^	200 W Kg^−1^		107
Ni–Al LDH/Ni-coated cotton textile	Electrolysis plating	Cotton cloth	1.18 mg cm^−2^	935.2 mF cm^−2^	1 mA cm^−2^	58.8 Wh kg^−1^	539 W kg^−1^	75.2% after 5000 cycles	159
G	DIW	Resorcinol and formaldehyde	4.2 wt%	85 F g^−1^	0.5 A g^−1^	4 kW kg^−1^		90% from 0.5 to 10 A g^−1^) and power densities	187
G\ethyl cellulose (EC)	Mixing inkjet-printed	Plastic Si Wafer	G (15 ppm), and 15 ppm ethyl cellulose	268 µF cm^−2^	10 mV s^−1^			97%, 1000	188
GO	Sprayed coating on Ti foil	Ti foil	GO (2 ppm)	132 F g^−1^	0.01 V s^−1^	6.74 Wh kg^−1^	2.19 kW kg^−1^	96.8%, 1000	189
	Thermal treatment								
MnO_2_	Inkjet printing	Polyimide film substrates	MnO_2_ (8.8 ppm)	2.4 F cm^−3^	10 m Vs^−1^	0.018 W cm^−3^		88% after 3600	207
NiO	Ink preparation inkjet-printed	PET	Ethylene glycol	155 mF cm^−2^	5 mV s^−1^	0.12 W cm^−3^		90%, 4000	211
			TRITON® X-100 surfactant	705 F cm^−3^					
V_2_O_5_	Multi-ink printing	MCNTs	V_2_O_5_ NWs/MWCNTs, PVA/KOH, VN NWs/MWCNTs, and PVA/KOH	59.3 F g^−1^	5 mV s^−1^	21.1 Wh kg^−1^	54.3 µWh cm^−2^	95.5% after 5000 cycles	213
Ti_3_C_2_T_*x*_/C12 × 10^9^	3D printing	PE		1.58 F cm^−2^	5 mV s^−1^	0.084 mWh cm^−2^	0.847 mW cm^−2^	10 000 cycles, 86.5%	219
d-Ti_3_C_2_T_*x*_	Sonication and coating	PET	72.3 wt%	2.337 F cm^−2^	2 mV s^−1^	207.81 µWh cm^−2^	3.74 mW cm^−2^	93.1%, 10 000	220
Ti_3_C_2_T_*x*_	Inkjet/extrusion-printed	MSC, AlO_*x*_-@PET	100%	562F cm^−3^	1 V s^−1^	0.32 µWh cm^−2^	11.4 µW cm^−2^	97–00%, 1000 cycles	221
Ti_3_C_2_	3D printing	Al foil, Cu foil, glass	Super P carbon black : LA132 : Binder at 8 : 1 : 1	70.1 mF cm^−2^	10 mV s^−1^	0.42 mWh cm^−2^		92%, 7000	224
Ti_3_C_2_T_*x*_ MXene	Exfoliation	Paper		108.1 mF cm^−2^ and 720.7 F cm^−3^	1 A g^−1^	100.2 mWh cm^−3^	1.9 W cm^−3^	94.7%, 4000	223
	Inkjet printing								
Ti_3_C_2_T_*x*_\MnO, Ag\C_60_	3D printing	MSC	MXene, AgNWs, MnO, and C_60_ with a weight ratio of 3 : 4:0.5 : 0.2	216.2 mF cm^−2^	10 mV s^−1^	19.2 µWh cm^−2^	58.3 mW cm^−2^	75%, 1000	225
Polymer		Polymers	Acrylate oligomer: acroleic acid: photoinitiator: dditives 70 : 25 : 2 : 2	0.65 mC m^−3^	10 mV s^−1^	10.98 W m^−3^			177

**Fig. 3:**
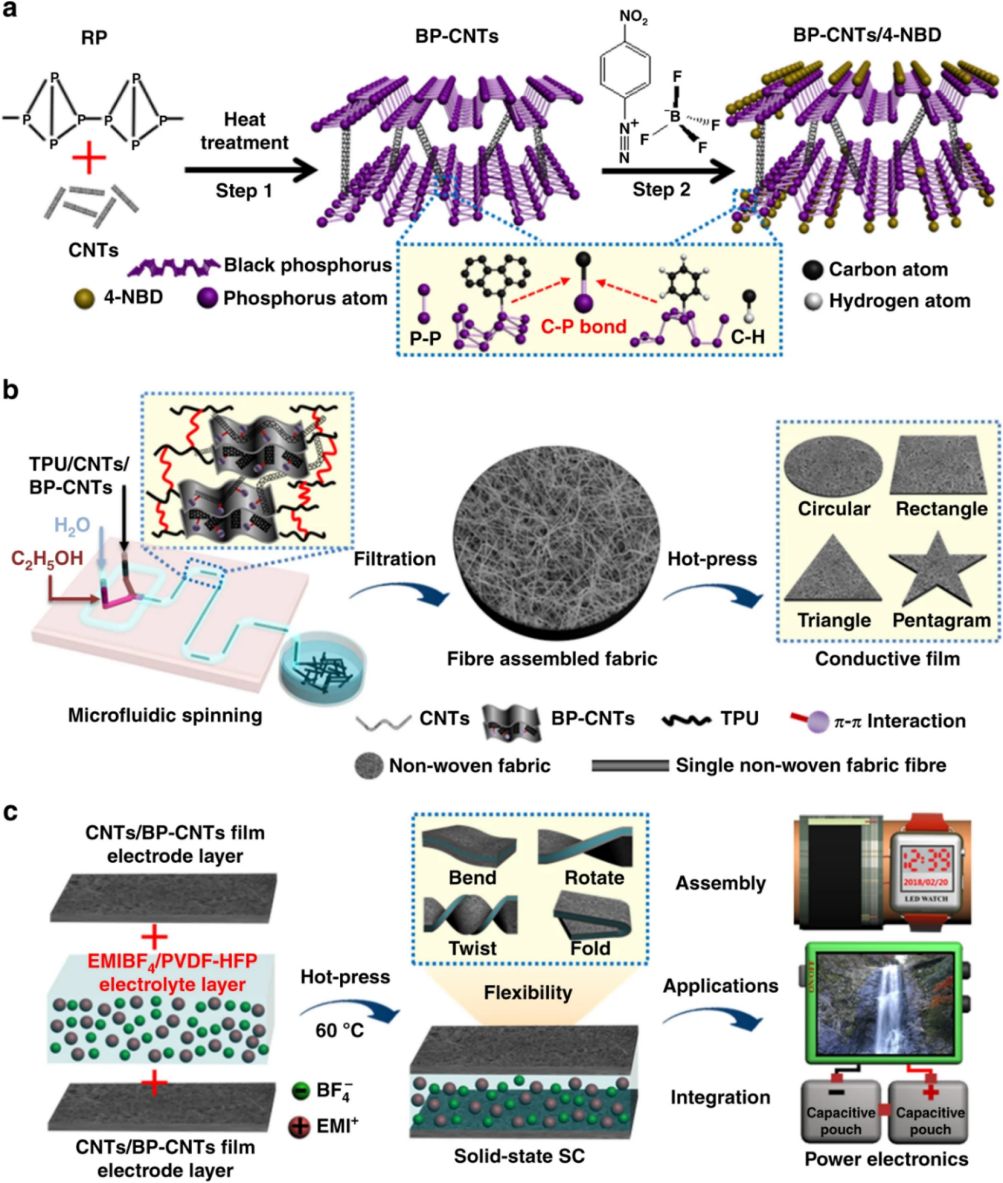
(a) Chemically bridged BP/CNT hybrid materials were synthesized through a heat-treatment process, followed by surface functionalization using 4-NBD; (b) flexible BP/CNT microfibers were fabricated using a triphase MST and subsequently assembled into interconnected nonwoven fiber fabrics with excellent mechanical flexibility; (c) FSCs were constructed by hot pressing conductive fabric electrodes together with a polymer-supported ionic-liquid electrolyte layer. Figure reprinted from Open Access ref. 71. Copyright © 2018, The Author(s).

A heterostructured black phosphorus-carbon nanotube (BP/CNT) composite was created and fabricated into nonwoven fiber fabrics for high-performance FCs electrodes (Fig. 3).^71^ The BP/CNT hybrid was synthesized using a mineralizer-assisted gas-phase transformation technique, in which red phosphorus, Sn, SnI_4_, and CNTs were encapsulated in evacuated silica tubes and subjected to thermal treatment under controlled heating and cooling conditions. In the reaction, red phosphorus transformed into layered black phosphorus, with CNTs integrated within the black phosphorus framework, resulting in a conductive and mechanically stable BP-CNT heterostructure. The BP/CNT composite was chemically modified with 4-nitrobenzene-diazonium (4-NBD) to enhance interfacial characteristics and dispersion stability. The primary BP-CNT material was exfoliated in acetonitrile *via* ultrasonication, thereafter underwent diazonium functionalization, and was repeatedly washed to yield modified BP/CNT nanosheets with improved processability. Subsequently, flexible nonwoven fiber textiles were produced utilizing a triphase microfluidic spinning process. The microfluidic device had a central channel and two sheath-flow channels. A homogeneous dispersion of 4-NBD-modified BP/CNTs, CNTs, and thermoplastic polyurethane (TPU) in dimethylacetamide functioned as the core flow, whereas ethanol and water served as the sheath flows for pre-coagulation and deep coagulation *via* solvent exchange. Continuous microfibers were produced within the microreactor, collected by filtration, and subsequently hot-pressed and dried to create linked nonwoven fiber textiles. Solvent-vapor-assisted fusion established strong interfacial connections between fibers, yielding mechanically robust and electrically conductive flexible films. The engineered materials comprised consistent microfibers with an average diameter of around 80 µm, demonstrating superior electrical conductivity (75.2 Ω m^−2^), substantial mechanical strength (Young's modulus of 313 MPa), and exceptional flexibility with a fracture elongation of 17.96%, surpassing numerous previously reported graphene- and CNT-based flexible electrodes. The tiny films could be twisted, stretched, and cut into various shapes without structural breakdown, showcasing remarkable mechanical resilience. The enhanced electrochemical performance of the flexible supercapacitor was achieved through the synergistic integration of black phosphorus and CNTs. The open 2D BP/CNT architecture provided numerous ion-transport channels with subnanometer pores, improved electrical conductivity, facilitated rapid ion diffusion, and provided robust mechanical stability. As a result, the constructed flexible supercapacitor exhibited a significant volumetric capacitance of 308.7 F cm^−3^, an energy density of 96.5 mWh cm^−3^, exceptional cycling stability, and remarkable deformation tolerance. Due to these characteristics, the device effectively powers wearable and portable electronics, including LEDs, smartwatches, and flexible screens, underscoring the considerable promise of BP/CNT fiber topologies for future wearable energy-storage systems.^71^ CNTs can be further modified chemically with other metal-based components. For example, MoS_2_-CNTs wherein CNTs are vertically integrated inside a MoS_2_ matrix through C–Mo covalent linkages.^72^ Leveraging the *in situ* vertical bridge, high-speed interlaminar conductivity, unobstructed ion-diffusion pathways, and ample pseudocapacitive reactivity, the MoS_2_/CNTs exhibit an exceptionally high capacitance of 5485 F g^−1^. Furthermore, the fully integrated solid-state FCs produced *via* direct-write printing exhibit 226 mWh g^−1^, 723 F g^−1^, and stable performance across high and low temperatures, making them suitable for powering wearable health-monitoring devices (Fig. 3).^72^

Overall, the integration of carbon-based materials represents a highly effective strategy for balancing flexibility and electrochemical performance in FSCs. Continued progress in hybrid electrode engineering is expected to accelerate the development of next-generation FSCs for applications in wearable electronics, electronic skin, robotics, and other flexible systems. Carbon nanostructure was integrated with TENGs and SCs, offering a flexible self-charging power system.^73^

Flexible and wearable electronics represent one of the most transformative technological advances of the past decade. They should offer high flexibility, lightweight design, and seamless integration of electronic technologies. Applications such as electronic textiles, rollable displays, active RFID (radio frequency identification) tags, electronic skins, implantable medical devices, internet-of-things (IoT),^74^ and internet-of-everything (IoE) systems^75,76^ exemplify this transition toward highly interconnected technologies. These applications require power sources that are not only lightweight and safe but also capable of maintaining stable performance under bending, twisting, stretching, and folding conditions.

Freestanding 3D carbon-based structures, such as carbon foams and aerogels, are exceptionally promising candidates for compressible SCs. Their interlinked porous structures and coarse surface morphology confer low density, superior hydrophilicity, and effective ion transport. Furthermore, the honeycomb-like configurations efficiently transfer mechanical stress from vertical to lateral orientations, thus reducing structural failure and facilitating elastic energy storage during compression. Given that compressive deformation is a predominant mechanical stress in FSCs, the suitable design of compressible electrodes with excellent electrochemical and mechanical stability is imperative. Advances in compressible carbon-based materials have focused on improving conductivity, structural integrity, and charge-storage capacity concurrently. Carbon nanocomposites were also investigated with other materials *e.g.*, MXene/CNT,^77^ MnO_2_/CNT,^78–80^ copper metals,^81,82^ and graphene-metallic textiles.^83^

### Metal oxides/hydroxides/sulfides/phosphides/selenides/carbides

Metal oxides,^84^ oxytelluride,^85^ metal tellurides,^86^ hydroxides, sulfides, phosphides, selenides, and carbides^87^ have garnered significant interest as pseudocapacitive electrode materials for high-performance supercapacitors owing to their diverse oxidation states, rapid surface redox processes, and high theoretical capacitance (Table 1). These materials substantially enhance charge storage *via* faradaic electrochemical processes, thereby facilitating higher energy densities than traditional carbon-based ELDCs. Metal oxides, including NiO/CuO,^88^ CoO/Co_3_O_4_,^89^ Mn_2_O_3_ ^90^ and ceramics,^91^ are intensively investigated for their superior electrochemical activity, affordability, and environmental compatibility.^92^ Single, bimetallic, and multimetallic oxides were reported.^92^ Cation-exchange and oxygen-vacancy-induced capacity in hierarchical α-Ni_1−*x*_Cu_*x*_MoO_4_@CC flexible electrodes for energy-storage applications.^93^ Metal oxides with oxygen deficiency enable high electrochemical performance for supercapacitors.^94^ They are typically manufactured by hydrothermal, solvothermal, sol–gel, electrodeposition, thermal oxidation, and chemical bath deposition methods. Although metal oxides exhibit excellent capacitance and commendable chemical durability, they often exhibit inadequate electrical conductivity, limited rate capability, and structural deterioration with extended cycling.

Metal hydroxides and layered double hydroxides (LDHs) have layered architectures that promote ion diffusion and reversible redox processes.^95^ These materials are generally synthesized *via* co-precipitation, hydrothermal growth, chemical precipitation, and electrodeposition techniques. Metal hydroxides exhibit remarkably high specific capacitance and rapid electrochemical kinetics; nevertheless, their practical use is limited by low electrical conductivity, structural instability, and poor cycling durability.

Metal sulfides and selenides, *e.g.*, (Ni,Co)Se_2_ ^96^ MoSe_2_/NiSe,^97^ have emerged as alternatives to metal oxides due to the substantial improvements in electrical conductivity and electrochemical activity achieved by sulfur incorporation.^98^ Common sulfide-based electrode materials are manufactured using hydrothermal techniques, sulfidation processes, chemical vapor deposition, and electrodeposition. These materials demonstrate exceptional electron transport, high energy density, and extensive redox chemistry. However, sulfides may undergo sulfur dissolution, volumetric expansion, and structural instability during prolonged cycling, adversely impacting electrochemical performance and longevity.

Metal phosphides are being extensively studied for their metallic conductivity and rapid charge-transfer kinetics. They are typically manufactured by phosphorizing metal oxides or hydroxides with phosphorus-containing precursors in inert atmospheres. Metal phosphides exhibit superior electrochemical activity, higher power density, and greater conductivity than traditional oxides. Nonetheless, their production often requires intricate techniques and may pose issues related to oxidation sensitivity, air instability, and phosphorus toxicity. Metal selenides demonstrate exceptional electrical conductivity and fast ion/electron transport due to the reduced electronegativity of selenium.^16^ These materials are often synthesized *via* hydrothermal synthesis, selenization, electrodeposition, and chemical vapor deposition. Metal selenides exhibit elevated capacitance and exceptional electrochemical performance; yet they may suffer from inadequate long-term stability, selenium toxicity, and high synthesis costs.

Hybrid and composite electrodes that integrate metal compounds with conductive carbon nanotubes, conductive polymers, MXenes, carbon cloth, or flexible textiles have been widely developed to address the limits of individual material systems.^99^ These hybrid architectures enhance electrical conductivity, ion diffusion, mechanical flexibility, cycling stability, and structural durability. Moreover, fabrication methods, including direct ink writing, screen printing, inkjet printing, electrodeposition, wet spinning, microfluidic spinning, and additive manufacturing, have facilitated the creation of flexible and wearable supercapacitor electrodes featuring controlled architectures, improved electrochemical properties, and multifunctional capabilities for next-generation energy storage applications.

### MXene

MXenes are a class of 2D transition-metal carbides/nitrides/carbonitrides that have attracted significant attention for supercapacitor applications due to their high electrical conductivity, hydrophilicity, stable surface chemistry, and superior electrochemical performance (Table 1).^100–102^ They are often produced from layered MAX phases characterized by the formula M_*n*+1_AX_*n*_, where M signifies an early transition metal (such as Ti, V, Mo, or Nb), A represents an A-group element (usually Al or Si), and X denotes carbon and/or nitrogen.^103,104^ MXenes are typically produced *via* selective etching of the A-layer with acidic fluoride solutions, resulting in layered structures terminated with –OH, –O, or –F, collectively referred to as T_*x*_. Among the many MXenes, Ti_3_C_2_T_*x*_ is the most thoroughly studied due to its metallic conductivity, which can reach approximately 9880 S cm^−1^, as well as its superior ion accessibility and rapid charge-transfer capability. MXenes exhibit exceptional pseudocapacitive properties owing to the reversible intercalation of electrolyte ions within their layered architectures, thereby facilitating high volumetric capacitance, superior rate capability, and prolonged cycling stability. Moreover, their solution processability facilitates the formulation of printable inks appropriate for modern fabrication techniques. MXene-based electrodes may be combined with conductive polymers, carbon nanomaterials, metal oxides, or hydrogels to mitigate nanosheet restacking, augment ion transport, and promote mechanical flexibility. Notwithstanding their exceptional electrochemical capabilities, problems such as oxidation instability, nanosheet agglomeration, and restacking effects pose substantial obstacles to practical applications, as they decrease accessible surface area and impede electrolyte access. As a result, further research emphasizes surface engineering, the development of hybrid nanocomposites, and scalable manufacturing techniques to enhance the long-term stability, conductivity, and mechanical strength of MXene-based flexible and wearable supercapacitors.

A pliable, wearable, wireless-charging power system that incorporates a piezoelectric ultrasonic array harvester (PUAH) with MXene-based solid-state supercapacitors (MSSSs) in a soft wristband configuration for sustainable applications.^105^ The MSSS is constructed using Ti_3_C_2_T_*x*_ nanosheet-embedded carbon cloth frameworks as electrodes and a poly(vinyl alcohol)/H_3_PO_4_ gel as the electrolyte, achieving a high energy density (58.74 Wh kg^−1^) and a long cycle life (99.37% over 10 000 cycles). A 2D stretchable piezoelectric array functions as a wireless charging module by integrating high-performance materials, enabling wireless power transmission *via* ultrasonic waves, achieving a power density of 1.56 W cm^−2^.^105^

MXene can be easily integrated into flexible substrates.^106^ They were conjugated with other materials, including metal oxide, polymer, *etc*.^103^ Composite membrane electrodes of bacterial cellulose//PANI/nitrogen-doped MXene, denoted as BC/PANI/N-MXene, was reported.^107^ Using a controlled freeze-polymerization method, PANI was synthesized within and on the surface of the BC nanofiber network, resulting in a distinctive 3D, interconnected, flower-like nanostructured conductive scaffold. Vacuum-assisted self-assembly facilitated the homogeneous deposition and secure anchoring of MXene nanosheets onto the BC/PANI scaffold. As the mass percentage of N-MXene increases, the specific capacitance initially increases and then decreases. The improved composite membrane, BC/PANI/N-MXene (3%), demonstrated a remarkable specific capacitance of 5525 mF cm^−2^ (635 F g^−1^) at a current density of 5 mA cm^−2^. At a current density of 20 mA cm^−2^, it maintained 61% of its original capacitance, suggesting its potential for energy storage applications. This research presents a method for improving the electrochemical efficiency of PANI-based energy storage materials.^107^

### Polymers

Organic polymers with redox-active functional groups enabled high electrochemical performance. They can be classified into several types and classification. Several examples were reported, including a melamine-based polymer,^108^ and polybenzoxazine rresin.^109^ N-containing polymers exhibit high electrochemical performance.^110^ Conjugated polymer-based hydrogel enabled flexible and self-healable electrodes for flexible supercapacitors.^111^

The synthesis of conjugated polymers (P1 and P2) for supercapacitor applications was reported.^112^ The materials were synthesized using a condensation procedure involving tetraphenylethene (TPE) with di-(TPE-2CHO) or tetra-carboxaldehyde (TPE-4CHO) derivatives and 1,5-diaminonaphthalene (1,5-DAN).^112^ The P1 and P2 polymers exhibited a spherical morphology, with particle diameters measuring 6.8 ± 1 µm and 0.97 ± 0.1 µm, respectively. P1 and P2 demonstrated extensive light absorption (200–466 nm), with corresponding band gaps of 2.3 eV and 2.4 eV, respectively. At a scan rate of 1 mV s^−1^, P1 and P2 exhibited specific capacitances of 274.8 F g^−1^ and 207.9 F g^−1^, respectively. The polymer is recyclable for 5000 cycles with less than 10% reduction in efficiency.^112^

### Metal–organic frameworks (MOFs)

MOFs are porous materials of metal ions coordinated to organic ligands, resulting in highly organized 3D structures.^113–116^ Owing to their remarkably high surface area, adjustable pore structure, modifiable chemical composition, and numerous active sites, MOFs have attracted significant interest for supercapacitor applications. The porous structure of MOFs promotes effective ion diffusion and electrolyte infiltration, while their customizable metal centers and organic linkers allow for the creation of materials with specific electrochemical characteristics. Numerous MOFs using transition metals such as nickel, cobalt, copper, iron, manganese, and zinc have been examined as electrode materials for supercapacitors, as these metals can undergo reversible Faradaic redox processes, thereby improving pseudocapacitive performance.^113^ MOFs can be used as precursors for the synthesis of metal oxides,^117,118^ sulfides, phosphides, selenides, *etc* embedded into carbon materials.^119^ A Zr-based MOF was reported for preparing metal phosphides on GO/MXene.^120^

Bimetallic MOF was reported for the synthesis of NiO/CuO-embedded carbon for supercapacitor.^121^ Synthesis of metal-based materials using MOF materials ensures the presence of carbon residual with and without heteratom, improving the material conductivity.^122^ Hybrid nanomaterials offer promising properties for use as electrodes in hybrid supercapacitors. Dyes (rhodamine B, RhB) encapsulated zeolitic imidazolate frameworks (RhB@ZIF-8) were synthesized and used as a precursor for synthesizing zinc oxide-embedded nitrogen-doped carbon (ZnO@N-doped C) *via* carbonization.^123^ The electrode of ZnO@N-doped C offered a high capacitance of 1200 F g^−1^ at a current density of 1 A g^−1^.^123^

### Covalent organic frameworks

COF-based materials are promising materials for SCs (Table 1).^124^ The synthesis method for COFs composites has been examined in the ref. 125. COF-based nanocomposites possess the characteristics of both COFs and nanoparticles, facilitating use in energy storage devices. COF-based composites demonstrate the benefits of both COFs and the accompanying nanomaterials. They exhibit enhanced electrical conductivity and superior electrochemical performance compared with pristine COFs.

COFs serve as electroactive materials for supercapacitor electrodes.^126,127^ They demonstrate numerous advantages, including a substantial specific surface area, adjustable pore size and structure, low weight, and active functional groups. A Schiff-based COF composed of 1,3,5-tris-(4-aminophenyl)triazine and 2,6-diformyl-4-methylphenol was synthesized using a solvothermal approach through a condensation reaction. They exhibit a specific capacitance of 354 F g^−1^ at 2 mV s^−1^. The materials demonstrated excellent cyclic stability, retaining 95% of their specific capacitance after 1000 cycles. The high performance of the produced COFs can be attributed to multiple factors, including a high specific surface area (651 m^2^ g^−1^), extensive π–π conjugation, and intrinsic microporosity.^128^

A COF with a specific surface area showed a specific capacitance of 546 F g^−1^ at 500 mA g^−1^ in an acidic solution.^129^ The solid-state device exhibited a capacitance of 92 mF cm^−2^ at 0.5 mA cm^−2^, with a power density of 98 µW cm^−2^, with high recyclability for 10 000 cycles.^129^ The COFs functional groups offered high chemical stability and enhanced electrochemical performance.^129^ NWNU-COF-1 was synthesized through the condensation reaction of melamine with 2,4,6-trichloro-1,3,5-triazine, resulting in the formation of –NH– linkages.^130^ The synthesis protocol is straightforward and necessitates inexpensive reagents. NWNU-COF-1 had a specific surface area of 301.149 m^2^ g^−1^ and a pore size of 1.41 nm. It demonstrated pseudocapacitance of 155.38 F g^−1^ at 0.25 A g^−1^, showing 100% capacitance retention after 20 000 ^130^. NWNU-COF-4, consisting of 2,4,6-trihydroxypyrimidine and trinitrophenol, offered 133.44 F g^−1^ at 0.3 A g^−1^ and a retention rate of 94% after 10 000 cycles.^131^

COFs are considered promising electrode materials for supercapacitors because of their tunable porosity, high surface area, ordered crystalline structures, and π-conjugated frameworks, which collectively enhance charge transport and ion diffusion. Conjugated COFs exhibit superior electrochemical performance due to extended electron delocalization; for example, a conjugated polymer synthesized from piperazine-1,4-dicarboxaldehyde and pyrrole in the presence of FeCl_3_ and acetic acid exhibited a capacitance of 571 F g^−1^ at 1.0 A g^−1^, an energy density of 15.4 Wh kg^−1^, and retained 80% of its capacitance after 9000 cycles at 6.0 A g^−1^.^132^ Low-dimensional COFs, particularly 2D COFs, provide abundant electroactive sites and efficient ion-transport pathways, and can be synthesized using solvothermal, self-assembly, surfactant-assisted, template-directed, and solid-supported growth methods. A solvothermally synthesized PI-COF delivered capacitances of 163 F g^−1^ at 0.5 A g^−1^ and 96 F g^−1^ at 40 A g^−1^ with an energy density of 35.7 Wh kg^−1^ and capacitance retention of 84.1% after 30 000 cycles,^133^ while a 2D Schiff-base COF prepared from 1,3,5-triformylphloroglucinol and 1,5-diaminonaphthalene achieved 379 F g^−1^ and maintained 75% of its capacitance after 8000 cycles.^134^ The excellent performance of these materials is attributed to π-conjugation, interconnected porosity, and enhanced ionic conductivity. Incorporation of redox-active groups such as carbonyls,^135^ nitrogen-containing heterocycles,^136^ sulfur-containing species,^137^ and radical functionalities^138^ further enhances pseudocapacitive behavior through reversible proton/electron transfer reactions.^139^ Ionic COFs (iCOFs), containing charged or ionizable groups such as sulfonates, silicates, and imidazolates, have also attracted considerable attention because of their improved ionic conductivity and electrochemical activity.^140^ These materials can be synthesized either from ionic monomers or through post-synthetic modification;^141^ for example, early spiroborate-linked iCOFs exhibited Li^+^-ion conductivity of 3.05 × 10^−5^ S cm^−1^.^142^ COFs are also attractive precursors for producing heteroatom-doped porous carbons through carbonization.^143,144^ Fluorine- and nitrogen-*co*-doped porous carbons derived from covalent triazine frameworks exhibited a capacitance of 326 F g^−1^ at 1 A g^−1^ with an energy density of 31.4 Wh kg^−1^ and excellent stability over 10 000 cycles,^145^ whereas N-doped porous carbon derived from triazine-based COFs exhibited a surface area of 801 m^2^ g^−1^ and a capacitance of 505 F g^−1^ at 0.5 A g^−1^ with 89% retention after 10 000 cycles.^146^ Metal-ion functionalization has also been used to improve conductivity and redox activity; for instance, manganese-modified THPP-PA-Mn conductive COFs exhibited a capacitance of 90.9 F g^−1^ and energy densities of 12.6 Wh kg^−1^ at 1.0 kW kg^−1^.^147^ In addition, hybridization of COFs with conductive carbon nanomaterials such as graphene, activated carbon, and CNTs significantly improves conductivity, surface accessibility, and mechanical stability. NH_2_-f-MWCNT@COFTTA-DHTA composites synthesized *via* solvothermal methods exhibited capacitances of 127.5 F g^−1^ and 98.7 F g^−1^ at 0.4 and 2 A g^−1^, respectively, outperforming the pristine COF and CNT components,^148^ while COF-300/oxidized MWCNT composites enhanced the mechanical and electrochemical properties of the electrodes.^149^ Flexible composite membranes formed by infiltrating COFs into CNT films achieved capacitances up to 425 F g^−1^ in H_2_SO_4_ electrolyte,^150^ and redox-active c-CNT@COF nanofibers enabled flexible energy-storage devices with enhanced conductivity and flexibility.^150^ Similarly, graphene/COF composites demonstrated excellent electrochemical performance; COFs/NH_2_-rGO composites achieved a capacitance of 533 F g^−1^ at 0.2 A g^−1^ in Na_2_SO_4_ electrolyte,^151^ phosphine-based porous polymer/rGO aerogels delivered energy densities of 33.3 Wh kg^−1^ with 88% retention after 12 000 cycles,^152^ and anthraquinone-based DAAQ-COFs/graphene aerogel electrodes exhibited capacitances of 378 F g^−1^ and energy densities of 30.5 Wh kg^−1^.^153^ These studies collectively demonstrate that COFs and their composites are highly promising materials for next-generation high-performance supercapacitors, owing to their tunable structures, high porosity, redox activity, enhanced conductivity, and compatibility with flexible and wearable energy-storage systems.

Metal oxide-based COFs have been identified as electroactive materials for supercapacitors. They demonstrate substantial capacity and high energy densities. The capacitance of metal oxide-based materials arises from the reversible faradaic redox reaction with the electrolytes. Metal oxides, such as Fe_3_O_4_, can serve as templates for the synthesis of hollow COFs. A template-directed synthesis approach was documented for fabricating hollow COF materials through the etching of a Fe_3_O_4_ template with HCl.^154^ The incorporation of metal oxides into COFs nanocomposites presents the potential for use as electroactive materials in supercapacitor electrodes.

### Flexible substrates

**Fig. 4:**
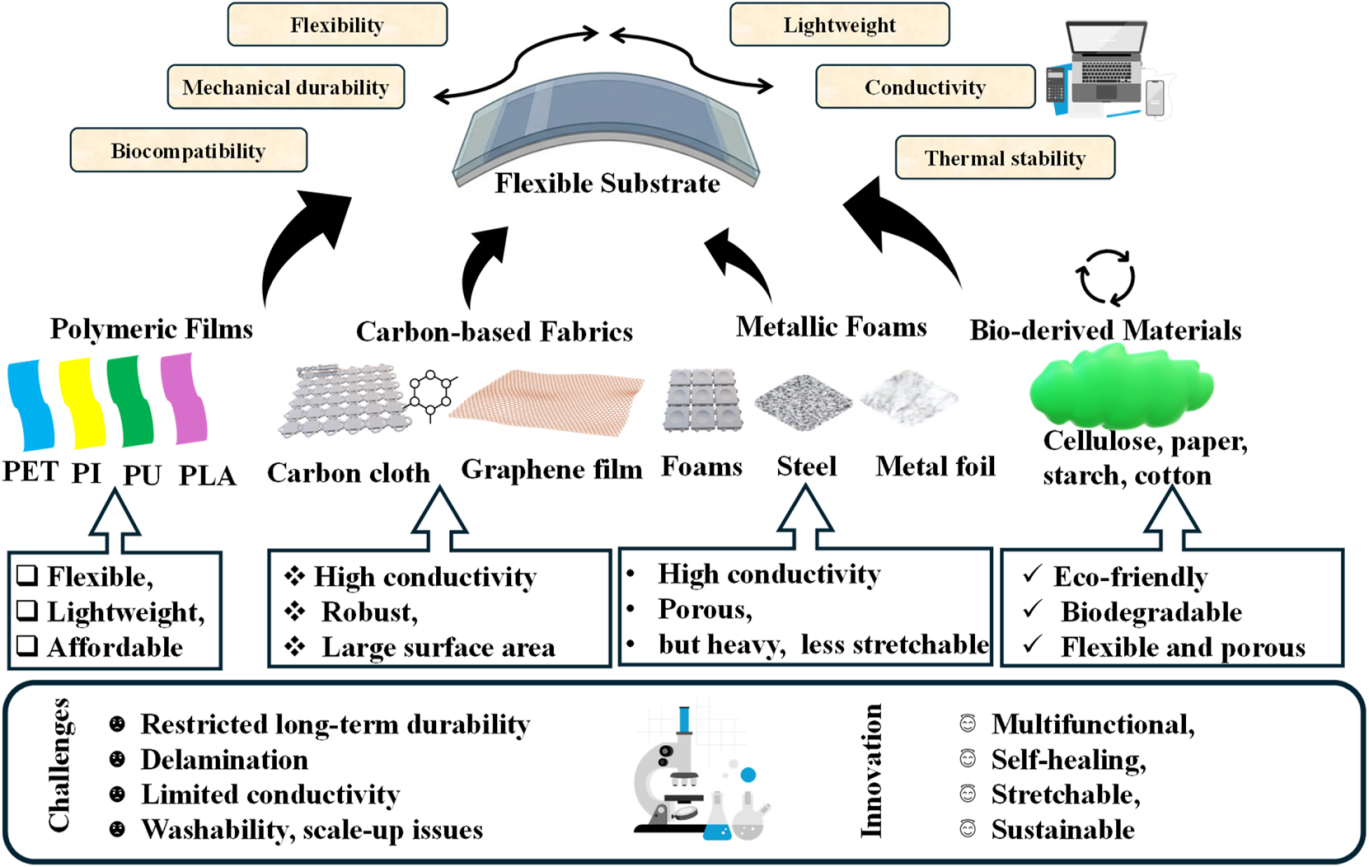
Flexible substrates showing types, advantages, challenges, and future directions.

Flexible substrates are crucial elements in wearable and deformable supercapacitors, as they offer mechanical support while preserving flexibility, lightweight properties, and structural integrity during mechanical deformation (Table 1). In contrast to traditional rigid substrates, flexible substrates enable energy-storage devices to withstand bending, folding, twisting, stretching, and compression without significant degradation in electrochemical performance (Fig. 4). The choice of a suitable substrate significantly affects the conductivity, mechanical durability, biocompatibility, flexibility, thermal stability, and overall efficacy of flexible supercapacitors (Fig. 4).

A diverse array of flexible substrates has been thoroughly examined, encompassing polymeric films, carbon-based fabrics, metallic foams, and sustainable bio-derived materials. Prevalent polymer substrates include polyethylene terephthalate (PET), polyimide (PI), and polylactic acid (PLA), owing to their affordability, lightweight characteristics, flexibility, and ease of processing (Fig. 4). Polymeric materials are widely used in printing processes, including screen printing, inkjet printing, direct ink writing, and 3D printing, to fabricate flexible electrodes and integrated supercapacitor systems. Many polymer substrates exhibit restricted electrical conductivity and heat resistance, frequently necessitating conductive coatings or hybridization with carbon nanostructures.

Carbon-based substrates, including carbon cloth, carbon fibers, G films, and CNT fabrics, are particularly appealing for their dual roles as flexible supports and conductive current collectors. These substrates have high electrical conductivity, extensive surface area, chemical stability, and mechanical durability, rendering them ideal for high-performance flexible and wearable supercapacitors. Moreover, metallic substrates such as nickel foam, stainless steel mesh, and ultrathin metal foils have been used for their high conductivity and porous architectures, which promote active material loading and electrolyte transport. Nonetheless, metallic substrates may experience increased weight and reduced stretchability and may also suffer from corrosion.

There has been a growing focus on environmentally friendly, biodegradable substrates sourced from natural resources, such as cellulose, paper, starch, cotton, and textile fibers. Cellulosic substrates offer benefits such as affordability, biodegradability, renewability, flexibility, porosity, and high electrolyte-absorption capacity (Fig. 4). Conductive textiles produced *via* coating, electroless plating, or the integration of conductive nanoparticles exhibit significant promise for wearable electronics and smart fabrics. Furthermore, starch- and paper-based substrates have surfaced as viable, eco-friendly options for flexible electronics due to their ability to be fabricated into transparent, conductive, and mechanically robust films. Advanced fabrication processes, including coating deposition, electrospinning, wet spinning, microfluidic spinning, laser processing, and additive manufacturing, have broadened the applications of flexible substrates in next-generation energy storage devices.^155^ Notwithstanding significant advancements, some challenges persist, including limited long-term mechanical durability, inadequate washability, interfacial delamination, insufficient conductivity, and challenges in large-scale production. Consequently, contemporary research endeavors focus on developing multifunctional, flexible substrates that exhibit improved conductivity, stretchability, self-healing properties, biocompatibility, and environmental sustainability for advanced wearable and self-powered electronic devices.

### Flexible thermoelectric electrode

Flexible thermoelectric electrode is promising and suitable for thermoelectric-based applications. Nitrogen-doped SWCNT/MXene thin film was designed for thermoelectric applications.^156^ It exhibits considerable progress, with a thermopower output of 14.11 µW cm^−2^ and a Seebeck coefficient of 1.61 mV K^−1^, even at a minimal temperature gradient of 40 °C. The results indicate a significant 32% improvement in thermoelectric performance, with a power output of 10.69 µW cm^−2^ for a Pt electrode under analogous conditions. This significant enhancement highlights the high efficiency and potential of thermoelectric applications, providing a feasible and economical alternative to conventional Pt-based solutions that offer effective thermoelectric energy harvesting, supported by the material's scalability and accessibility.^156^

### Flexible electrodes of carbon cloth

Carbon cloth (CC) serves as a potentially flexible substrate for fabricating flexible electrodes.^157,158^ Nonetheless, commercial carbon composites are hindered by elevated costs, substantial dead weight and volume, and limited electrochemical activity, significantly affecting the energy and power densities of EES. A porous CC (PCC) cathode and a hard CC (HCC) anode were used for as scalable and renewable cotton fabric. The microporous architecture of PCC guarantees a fully self-supporting structure, extensive specific surface area, and superior performance predicated on PF_6_^−^. The non-porous architecture, including localized graphitic nanodomains in HCC, facilitates effective sodium storage, comparable to that of a capacitor, while offering enhanced flexibility. As a result, both the PCC cathode and the HCC anode exhibit high reversible capacity, exceptional rate capability, and extended cycling life in half- and full-cell sodium-ion capacitor systems. A flexible all-cloth sodium ion capacitor is constructed using a PCC cathode, an HCC anode, and a cotton cloth separator, which ensures consistent power output even under bending and cutting, owing to its entirely woven structure.^157^

### Electrodes based on cotton/cotton textile

Wearable electronics have garnered considerable interest in recent years, resulting in a robust demand for flexible, lightweight, and conductive materials for energy storage devices. This study presents a straightforward, economical, and scalable technique for transforming standard insulating cotton fabric into a highly conductive nickel-coated cotton textile (NCT).^159^ Cotton fabric, cut from a T-shirt into 4 × 6 cm^2^ pieces, was treated with a sensitizing solution comprising SnCl_2_ and HCl and subsequently rinsed with deionized water. The fabric was subsequently submerged in an activation solution comprising PdCl_2_ and HCl to facilitate the surface preparation for metal deposition. Following activation, the textile was immersed in an electroless nickel plating bath containing NiSO_4_, NH_4_Cl, sodium citrate, and NaH_2_PO_4_ for 2 hours at pH 9–10. The material was separated and dried to achieve nickel-coated cotton. To enhance electrical conductivity, electrodeposition was carried out using Ni-coated cotton as the cathode and a platinum plate as the anode in a NiSO_4_/NH_4_Cl electrolyte. A steady voltage of 2.0 V was applied for 8 minutes, followed by washing and vacuum drying. To assess its appropriateness for wearable supercapacitors, low-crystalline Ni–Al layered double hydroxide (NiAl-LDH) nanoparticles were electrodeposited onto the conductive cloth. The NCT@NiAl-LDH electrode exhibited outstanding electrochemical performance, characterized by a specific capacitance of 935.2 mF cm^−2^, commendable rate capability, and robust cycle stability. The constructed asymmetric supercapacitor (ASC) attained an energy density of 58.8 Wh kg^−1^ (134 µWh cm^−2^) at a power density of 539 W kg^−1^ (1228 µW cm^−2^). The results indicate the significant potential of nickel-coated cotton textiles as flexible current collectors for wearable energy storage applications.^159^

### Electrodes-based on starch

Starch is a suitable biodegradable material for flexible electronics due to its ability to form films readily by heating in water, followed by casting and drying.^74^ Nonetheless, films composed entirely of starch are typically fragile and exhibit poor transparency. To address these limitations, starch is frequently blended with other polymers or plasticizers or modified through physical and chemical treatments to enhance its mechanical strength, flexibility, conductivity, and stability. These enhancements render starch-based materials suitable for flexible electronic substrates and energy-storage applications. Starch-based conductive films are often fabricated by integrating electrically conductive substances, including graphene, CNTs, conductive polymers, ionic liquids, metals, or metal alloys. Starch nanocrystals (SNCs), generated by acid or enzymatic extraction of the amorphous portions of starch, have been used to improve composite performance. Surface nanocomposites enhance mechanical strength, biodegradability, and filler distribution in conducting films. Conventional starch-film fabrication often entails gelatinization and solution casting with plasticizers such as glycerol, ethylene glycol, citric acid, or ionic liquid 1-ethyl-3-methylimidazolium acetate ([C_2_mim][OAc]). In addition to film casting, starch is extensively used in conductive inks for printed electronics. Starch has become a significant material in supercapacitor applications due to its fulfilment of the “3E” criteria for energy-storage materials: ecologically sustainable, cost-effective, and readily processable.

Starch-based polymer electrolytes have demonstrated high electrochemical performance. Chen *et al.* created ultrathin carbon nanosheet-supported nickel quantum dot hybrids by starch-assisted hydrothermal synthesis.^160^ The supercapacitor demonstrated a specific capacitance of 1120 F g^−1^ at 2 A g^−1^, with 97% capacitance retention after 2000 cycles, underscoring starch as a cost-effective and sustainable carbon source for energy storage.^160^ Owing to its high carbon content, affordability, and availability, starch has emerged as a significant precursor for porous carbon electrode materials in supercapacitors.^161,162^ Wang *et al.* synthesized flexible 3D porous graphene electrodes using a combination of starch and graphene oxide, which were then carbonized and activated. The electrodes demonstrated a specific surface area of 1519 m^2^ g^−1^, an energy density of 19.8 Wh kg^−1^, and remarkable cycling stability with 80% capacitance retention after 8000 cycles.^163^ Liu *et al.* synthesized linked porous carbon electrodes from cornstarch, achieving a specific capacitance of 372 F g^−1^ in KOH electrolyte.^162^ Furthermore, Willfahrt *et al.* created printable starch/citric acid hydrogel electrolytes exhibiting an ionic conductivity of 2.30 ± 0.07 mS cm^−1^ and exceptional printability, facilitating the production of printed supercapacitors with a capacitance of up to 54 F g^−1^.^164^

Starch-derived materials have been utilized in TENGs for autonomous wearable devices. TENGs produce electricity *via* triboelectric and electrostatic induction effects between materials possessing distinct triboelectric characteristics. Bao *et al.* created lignin-starch composite TENGs, in which starch enhanced film homogeneity and device efficacy.^165^ Starch-film-based TENGs were reported for autonomous sweat detection by utilizing the interaction between human skin and starch films.^166^ They offered economical starch-based TENGs.^167,168^ Sarkar *et al.* created a thermoplastic starch/PDMS bilayer TENG that can produce open-circuit voltages of over 560 V and current densities of around 120 mA m^−2^, adequate to power over 100 LEDs.^169^ The device furthermore operated as a self-sustaining pedometer and gait-analysis sensor.

### Processing methods used for electrode fabrication

The advancement of advanced fabrication processes for flexible supercapacitor electrodes, characterized by small size, enhanced flexibility, lightweight composition, and superior deformability, has gained paramount significance for next-generation wearable and portable electronics.^170,171^ Significant research has focused on developing scalable, adaptable manufacturing methods that can yield mechanically robust electrodes while preserving superior electrochemical performance. The most extensively investigated approaches include coating deposition techniques, on-chip writing procedures, solution casting, wet-spinning, and dry-spinning, all of which provide uniform material dispersion and controlled electrode designs. Methods of coating deposition, such as spray coating, dip coating, electrophoretic deposition, and spin coating, are frequently used to produce thin, uniform conductive coatings on flexible substrates. These technologies provide precise control of film thickness and active-ingredient loading, while ensuring compatibility with large-scale, cost-effective production. On-chip writing and 3D printing, including direct-writing methods such as inkjet printing, extrusion printing, and laser writing, offer significant benefits by facilitating the accurate patterning of microscale electrode structures appropriate for integrated and downsized supercapacitor systems.^172^ These methods are especially appealing for flexible micro-supercapacitors since they enable adjustable electrode configurations, minimize material waste, and ensure compatibility with wearable electronic devices.

Solution-casting techniques have been thoroughly examined due to their simplicity and scalability. This method involves dispersing electroactive compounds in a solvent mixture and casting them onto flexible substrates to create free-standing or substrate-supported films. Wet-spinning and dry-spinning methods enhance the variety of electrode structures by generating continuous conductive fibers and yarns suitable for direct incorporation into textile-based energy-storage systems. Wet-spinning typically uses coagulation to create aligned, conductive fibers from liquid dispersions, whereas dry-spinning obviates the need for coagulation baths, enabling the direct production of lightweight, conductive fibers with enhanced structural integrity. Alongside traditional fabrication processes, numerous new manufacturing techniques have recently surfaced for the creation of flexible electrodes. Electrospinning has garnered significant interest due to its ability to produce ultrafine nanofibrous membranes characterized by interconnected porosity, extensive surface area, and superior electrolyte accessibility.^155^ Likewise, freeze-drying and template-assisted assembly techniques have been utilized to fabricate hierarchical porous aerogels and three-dimensional conductive networks that promote swift ion transport and mechanical flexibility. Vacuum filtration and layer-by-layer assembly methods are extensively employed to produce thin, free-standing films with highly organized structures and adjustable thicknesses. Moreover, 3D printing technologies, such as direct ink writing (DIW), inkjet printing, stereolithography, and extrusion-based printing, have enabled the production of customized, intricate electrode geometries with controlled porosity, mechanical integrity, and electrochemical performance.

Notwithstanding these advancements, numerous problems continue to constrain the efficacy of flexible electrodes. In numerous instances, insufficient miscibility and inadequate interfacial compatibility among components during fabrication procedures lead to structural inhomogeneity, aggregation, and weak interfacial adhesion. These difficulties can markedly reduce electrical conductivity, impede ion and charge transport at interfaces, and undermine the achievable energy density and mechanical stability of the electrode systems. Moreover, continuous bending, stretching, and deformation may cause structural damage or delamination, therefore impairing long-term electrochemical performance. Thus, there is a significant need to develop optimized fabrication techniques and hybrid material systems that can concurrently enhance conductivity, interfacial charge transport, mechanical durability, and electrochemical energy-storage performance in flexible supercapacitor electrodes.

### 3D printing of flexible electrodes

Among several reported methods,^173^ 3D printing can be achieved *via* several methods, including DIW,^174^ fused deposition modeling (FDM, fused filament fabrication (FFF)), stereolithography (SLA), and powder-bed selective laser sintering (SLS),^175^ and vapor printing.^176^ 3D printing enabled several configurations, including sandwich, interdigitated, fiber-shaped, integrated arrays, *etc.* 3D printing enables ultra-flexible TENGs.^177^

DIW is considered the predominant 3D-printing technology for EES devices due to its versatility in material selection, high-throughput production potential with multi-nozzle systems, and its ability to create bespoke free-standing structures with intricate geometries.^178,179^ In DIW, a viscous ink or slurry is extruded through a nozzle utilizing a specialized printing apparatus. DIW facilitates the rapid construction of complex three-dimensional structures by sequentially depositing materials along the *z*-axis, enabling precise microstructural control, high active-material loading, and improved energy production within compact device dimensions. The adaptability and scalability of DIW have prompted significant investigation into various energy-storage systems, including flexible batteries,^180,181^*e.g.*, LIBs, lithium–sulfur batteries (LSBs), zinc-ion batteries (ZIBs), and SCs.^182^ The porous scaffold increased surface area and promoted effective electrolyte infiltration, leading to superior capacity and prolonged cycling stability in lithium-ion batteries. Notwithstanding these substantial advancements, numerous challenges persist for DIW-printed energy-storage systems. Thick 3D-printed electrodes often exhibit high interlayer resistance due to the layer-by-layer deposition method, which can impede electron transport and reduce active material utilization. Moreover, ion diffusion within intricate 3D structures is often hindered by convoluted transport channels, thereby adversely affecting electrochemical kinetics. Post-processing procedures, including solvent evaporation and thermal treatment, may yield loose, highly porous structures, resulting in comparatively low volumetric energy density. The mechanical robustness of DIW-printed electrodes is limited by the inherent properties of printable materials, underscoring the need for improvements in structural integrity, conductivity, and multifunctionality for future energy-storage applications.

### 3D printing of polymers

Conductive polymers (CPs) are widely studied as electroactive materials for SCs electrodes owing to their high electrical conductivity and pseudocapacitive charge-storage characteristics. Common CPs, such as PANI and polypyrrole (PPy), undergo rapid and reversible redox processes, making them suitable for high-performance EES devices. Advances in manufacturing techniques, especially 3D printing, have enabled the creation of intricate polymer structures with tunable porosity, morphology, and active-material content, thereby improving the electrochemical performance of CP-based electrodes.^183^ Printing techniques enable the incorporation of conductive organic polymers and COFs into flexible and customizable supercapacitor devices.

Notwithstanding these benefits, CPs continue to experience numerous constraints, including inadequate cycling stability, diminished mechanical strength, and modest intrinsic conductivity. To mitigate these limitations, CPs are frequently integrated with carbon-based nanomaterials, including graphene, CNTs, and carbon black, which enhance conductivity, structural integrity, and electrochemical stability. These hybrid solutions demonstrate superior mechanical durability and electrochemical efficacy relative to unmodified conductive polymers.

A variety of thermoplastic polymers have been investigated for 3D printing applications, including acrylonitrile butadiene styrene (ABS),^184^ polyimide (PI),^185^ and polylactic acid (PLA).^186^ These polymers are often combined with conductive carbon nanostructures to enhance electrical conductivity and mechanical flexibility. These polymer–carbon composites are promising for producing flexible supercapacitor electrodes. Nonetheless, commercially available conductive filaments often contain only small amounts of electroactive fillers. For instance, commercial PLA/graphene filaments have merely 8% graphene, leading to inadequate electrical percolation and constrained electrochemical performance post-printing. Thus, enhancing the incorporation and distribution of conductive nanoparticles inside printed polymer matrices continues to pose a significant challenge.^186^

### 3D printing of carbon materials

Graphene is a monolayer 2D carbon material characterized by low density, remarkable mechanical properties, thermal stability, extensive surface area, and superior electrical conductivity. Carbon nanomaterials, such as AC, carbon black, G, GO, rGO, and CNTs, are widely used as electrode materials for SCs due to their high electrical conductivity, large surface area, affordability, and structural versatility. Diverse 3D-printing techniques have been established to fabricate these materials into SC designs.

3D printing *via* DIW of graphene-based materials enabled the fabrication of a self-supporting microlattice supercapacitor electrode.^187^ 3D graphenes exhibit reduced physicochemical properties relative to their 2D counterparts due to their singular composition and convoluted, random porosity. These constraints can be mitigated by creating a graphene composite material with a designed porosity structure. The 3D-printed graphene composite aerogel (3D-GCA) electrodes are lightweight, highly conductive, and demonstrate superior electrochemical characteristics. Specifically, the supercapacitors employing these 3D-GCA electrodes, with thicknesses in the millimeter range, exhibit remarkable capacitive retention (approximately 90% from 0.5 to 10 A g^−1^) and power densities exceeding 4 kW kg^−1^, comparable to or surpassing those of previously reported devices utilizing electrodes 10–100 times thinner. This study exemplified how 3D-printed materials, including graphene aerogels, can considerably expand the design possibilities for manufacturing high-performance, fully integrable energy storage devices tailored for diverse applications.^187^ Graphene-based micro-supercapacitors (MSCs) have been produced *via* self-aligned capillarity-assisted lithography for electronics (SCALE), which relies on the self-aligned deposition of liquid inks within microfluidic channels on plastic substrates.^188^ This technique enables scalable production with superior structural integrity and is especially appropriate for compact portable electronic devices.

Inkjet printing (IJP) has been widely employed for GO-based supercapacitor electrodes.^189^ GO exhibits superior water dispersibility and hydrophilicity, facilitating the formulation of stable and uniform inks. In a work, water-based GO ink was spray-coated onto titanium foil and then thermally reduced at 200 °C under a nitrogen atmosphere to produce conductive rGO electrodes.^189^ This method facilitated the production of flexible interdigital electrode arrays with lateral resolutions of about 50 µm and advantageous electrochemical capacitance. Printable electrodes based on CNTs have been similarly fabricated using thermoplastic polyimide (TPI) filaments with CNT loadings ranging from 1 to 9 wt%. The progressive increase in CNT concentration led to a reduction in electrical resistivity, with a conductive percolation threshold identified at around 3 wt% CNT loading.^185^

Hybrid carbon/polymer systems have exhibited superior electrochemical performance. A composite was created by 3D printing COF-derived microporous carbon (MPC) nanoparticles coated with PPy onto carbon sheets, followed by annealing in an argon atmosphere.^190^ The MPC framework offered a bimodal porous architecture and elevated surface area, however the PPy coating markedly improved capacitance, attaining a specific capacitance of 2.55 F cm^−2^.^190^

Carbon-based printable inks have also been reported. A hybrid GO ink comprising 98% GO and 2% commercial pen ink was effectively utilized for printed SC electrodes.^191^ Another method involved the impregnation of porous carbon compounds into cotton and polyester fabrics to produce flexible textile-based electrodes exhibiting high electrochemical capacitance.^192^

Hybrid materials are essential in supercapacitors because they integrate EDLC with pseudocapacitive charge-storage mechanisms, thereby enhancing overall electrochemical performance. A variety of hybrid material classes have been engineered for supercapacitor electrodes, encompassing carbon–carbon composites like AC/CNTs,^193^ carbon–metal nanoparticle systems such as GO/silver (Ag) and multiwalled CNTs/Ag nanoparticles,^194,195^ carbon–conductive polymer composites including graphene/PANI,^196^ and carbon–metal oxide nanocomposites^197,198^ like AC/manganese dioxide,^199^ GO/nickel hydroxide,^200^ single-walled CNTs/ruthenium dioxide,^201^ and graphene/vanadium dioxide (VO_2_).^202^ Fe_2_O_3_/CNT composites have been effectively produced *via* screen-printing methods on carbon-cloth substrates.^203^ The printed electrodes were later sintered using a nitrogen atmospheric-pressure plasma jet (APPJ) with a direct-current pulse configuration. This treatment efficiently eliminated residual solvents, enhanced surface wettability, and reduced carbon damage. The inclusion of 5 wt% CNTs markedly increased the specific capacitance from 16 to 54 F g^−1^.^203^ A nanocomposite including SnO_2_, CNTs, ethyl cellulose, and terpineol was synthesized utilizing the identical 3D-printing and APPJ-assisted processing technique.^204^ The resultant nanoporous electrode had a significantly elevated specific capacitance of 188.42 F g^−1^ at a scan rate of 2 mV s^−1^.^204^ These investigations demonstrate that nitrogen APPJ processing is a proficient method for generating nanoporous hybrid metal oxide electrodes with improved electrochemical performance for advanced supercapacitor applications.

### Metal oxides and chalcogenides in fabricated supercapacitors

Metal oxides represent attractive pseudocapacitive materials because to their high theoretical capacitance and affordability.^205^ Layered δ-MnO_2_ nanosheets were organized into Langmuir–Blodgett (LB) films on silicon and polyimide substrates.^206^ Before deposition, the substrates were treated with oxygen plasma to enhance adhesion. The resultant all-solid-state symmetric MSCs demonstrated a volumetric capacitance of 2.4 F cm^−3^ and a power density of 0.018 W cm^−3^, while preserving 88% capacitance retention after 3600 charge–discharge cycles.^207^ Furthermore, hybrid systems based on metal oxides and chalcogenides, like CuS/MXene^208^ and manganese hexacyanoferrate (MnHCF)/MnO_*x*_,^209^ carbon fiber (CF)/metal-oxide,^210^ have exhibited notable electrochemical properties.

Inkjet printing has been utilized to manufacture NiO-based MSCs.^211^ This method employed ethylene glycol as the solvent, in conjunction with the TRITON^®^ X-100 surfactant, to formulate printable inks. The resultant thin-film NiO electrodes exhibited electrical conductivity nearly 14 times greater than that of single-crystal NiO. Using magnesium perchlorate as an aqueous electrolyte, the devices operated over a wide voltage range of 1.5 V, achieving areal and volumetric capacitances of 155 mF cm^−1^ and 705 F cm^−1^, respectively.^211^ These printable MSCs exhibit significant possibilities for digitally designed and scaled printed electronics. Metal chalcogenides have also been investigated for printed superconducting devices. A flexible thin-film electrode composed of NiCo_2_O_4_ and NiCo_2_S_4_ powders co-precipitated onto Ni-grid substrates exhibited notable electrochemical performance for affordable wearable energy storage applications.^212^

**Fig. 5:**
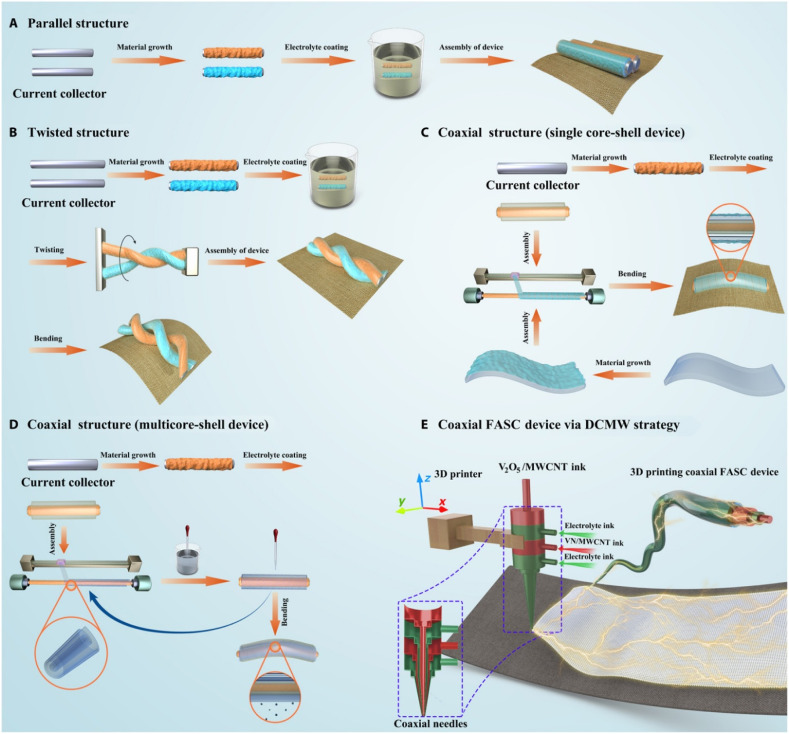
Schematic illustration of the fabrication processes for various FASC devices. The figure compares the preparation strategies of conventional FASC configurations, including (A) parallel, (B) twisted, and (C and D) coaxial architectures, with (E) the developed 3D-printed coaxial FASC fabricated using DCMW technology. Figure reprinted from the Open Access ref. 213 Copyright © 2021, The American Association for the Advancement of Science.

A coaxial fiber-shaped ASC (FASC) was effectively produced *via* a direct coherent multi-ink 3D-printing method (DCMW) (Fig. 5).^213^ This method relied on engineering the internal configuration of coaxial printheads, enhancing the rheological properties of the inks, and regulating the feed rates of various materials during printing. The device used V_2_O_5_ nanowires (VN) as the anode and VN nanowires as the cathode due to their high theoretical capacitance and wide electrochemical potential window. The manufacturing technique used a custom multicore-shell printer to enable the concurrent extrusion of electrode and electrolyte inks. The printable inks comprised V_2_O_5_ nanowires/MWCNTs, VN nanowires/MWCNTs, and a poly (vinyl alcohol)/potassium hydroxide gel electrolyte. Their formulations were modified to achieve viscous, shear-thinning, non-Newtonian characteristics, thereby ensuring smooth extrusion and structural integrity during printing. The viscosity of the electrolyte ink was deliberately reduced while remaining compatible with the electrode inks to ensure synchronized printing. After printing, the coaxial FASC structure was swiftly solidified in a PVDF/NMP coagulation bath, followed by solvent extraction and drying to maintain the device morphology. The integration of MWCNTs enhanced ink conductivity, viscosity, and mechanical strength. Rheological analyses demonstrated pronounced shear-thinning behavior, making the inks well-suited for extrusion-based 3D printing (Fig. 5). The small coaxial design offered substantial mass loading, effective ion transport, exceptional areal energy and power densities, together with outstanding mechanical flexibility and stability. Due to these properties, the printed FASC device was effectively combined with mechanical components and pressure sensors, showcasing its potential for self-powered wearable electronics, monitoring systems, and multipurpose energy-storage applications.^213^

### Three-dimensional printing of MXene-Based supercapacitors

MXenes exhibit remarkable characteristics for supercapacitor applications, including as high electrical conductivity, hydrophilicity, mechanical flexibility, and the capacity to intercalate ions within their layers.^214–217^ Ti_3_C_2_T_*x*_ demonstrates an electrical conductivity of up to 9880 S cm^−1^.^216^ These characteristics render MXenes exceptionally promising materials for flexible and printable energy-storage systems.^218^

**Fig. 6:**
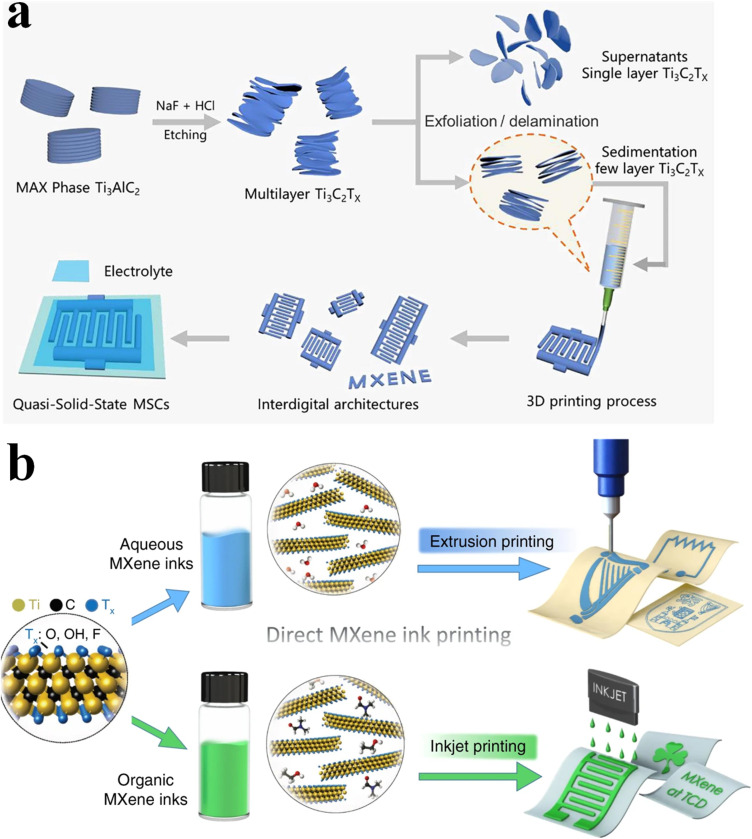
Schematic illustration of three-dimensional (3D) printing of (a) symmetric micro-supercapacitors (MSCs) with interdigitated electrodes using Ti_3_C_2_T_*x*_ MXene sediment inks, together with (b) direct MXene ink printing based on Ti_3_C_2_T_*x*_ organic inks for flexible energy-storage applications. Figure reprinted with permission from ref. 220 and 221 (Open Access, licensed under the Creative Commons Attribution 4.0 International License).

The manufacture of MXenes typically entails etching MAX phases with powerful chemical solutions, such as sodium fluoride and hydrochloric acid, followed by washing and freeze-drying. Surfactants, such as nonaethylene glycol monododecyl ether, have been employed to inhibit restacking and facilitate hydrogel formation, thereby establishing interconnected 3D networks that enhance ion accessibility.^219^ Direct 3D printing of aqueous Ti_3_C_2_T_*x*_ MXene sediment inks devoid of additives has been documented for the creation of quasi-solid-state MSCs (Fig. 6a).^220^ MXene sediment ink, mixed in DMSO/water, was printed onto PET substrates to create interdigital electrode patterns. The resulting devices exhibit areal capacitance of 2.337 F cm^−2^, an areal energy density of 207.81 µWh cm^−2^, and power density of 3.74 mW cm^−2^, while maintaining 93.1% capacitance after 10 000 cycles.^61^ The extrusion and inkjet printing of Ti_3_C_2_T_*x*_ organic inks have been demonstrated as well (Fig. 6b).^221^ Flexible MSCs with a volumetric capacitance of 562 F cm^−3^ and an energy density of 0.32 µWh cm^−2^ were created by regulating the quantity of organic solvents such *N*-methyl-2-pyrrolidone (NMP).^221^ MXene inks can be conjugated with other substances, like graphene, Prussian Blue, or conductive polymers, to enhance electrochemical performance.^222^ MXene dispersions, resistant to oxidation and produced with sodium ascorbate (SA), demonstrated exceptional stability in aqueous environments for more than 80 days without considerable aggregation.^223^ The ascorbate molecules reduced oxidation resistance and promoted ion transport *via* hydrogen-bonding interactions with MXene layers, resulting in greater charge-storage capacity.^223^

Nonetheless, MXene-based printed electrodes suffer from layer restacking caused by van der Waals interactions. Restacking reduces available surface area and impedes diffusion of electrolyte ions, thereby impairing electrochemical performance. Strategies including the integration of polymer microspheres and the development of porous structures have been investigated to mitigate these challenges.^224^ Stretchable MXene-based supercapacitors have also been produced. A printable nanocomposite gel including Ti_3_C_2_T_*x*_ MXene nanosheets, MnO_2_, silver nanowires, and fullerene was synthesized using unidirectional freezing. The resultant honeycomb-like porous MSCs demonstrated an areal capacitance of 216.2 mF cm^−2^, an energy density of 19.2 µWh cm^−2^, and a power density of 58.3 mW cm^−2^.^225^ Nonetheless, the devices retained approximately 50% of their functionality after 1000 cycles, underscoring the need for further improvements in cycling durability.

### Three-dimensional printing of MOFs-Based supercapacitors

MOFs are porous hybrid materials characterized by highly adjustable architectures and remarkably high surface areas. DIW was reported to fabricate 2D MOFs such as M (Cu, Cu/Co, or Cu/Ni)-tetrahydroxy-1,4-quinone on CNT/rGO aerogel electrodes.^226^ Direct laser scribing has been utilized to produce core–shell MOF composites, including MOF-199@ZIF-67.^227^ After carbonization, extremely porous carbonaceous electrodes featuring hierarchical topologies were produced. The printed MSCs exhibited rapid ion transport and high capacitance owing to their interconnected porous structures and well-organized mesopores. At a scan rate of 1 mV s^−1^, the devices attained areal and volumetric capacitances of 8.1 mF cm^−2^ and 6.0 F cm^−3^, respectively.^227^ The electrochemical performance was significantly influenced by the MOF-199-to-ZIF-67 ratio, underscoring the need for structural optimization.

Alongside SC electrodes, advanced 3D printing technologies have enabled the production of energy-harvesting devices, including TENGs and Zn–Co batteries.^228^ A highly adaptable three-dimensional triboelectric nanogenerator, created using hybrid UV-assisted 3D printing, has recently been developed for wearable electronics and IoT applications.^141^ The apparatus employed printed photopolymer resin configurations and ionic hydrogels as triboelectric layers and electrodes. Due to its designed 3D geometry and Maxwell displacement-current-driven mechanism, the TENG attained a peak power density of 10.98 W m^−3^ and a charge density transfer of 0.65 mC m^−3^ at a low operating frequency of around 1.3 Hz.^141^ The system effectively powers wearable and portable electronics, including smart shoes, temperature sensors, SOS signalling devices, and smartwatches, underscoring the potential of hybrid 3D-printed self-powered systems for future wearable technologies.

**Fig. 7:**
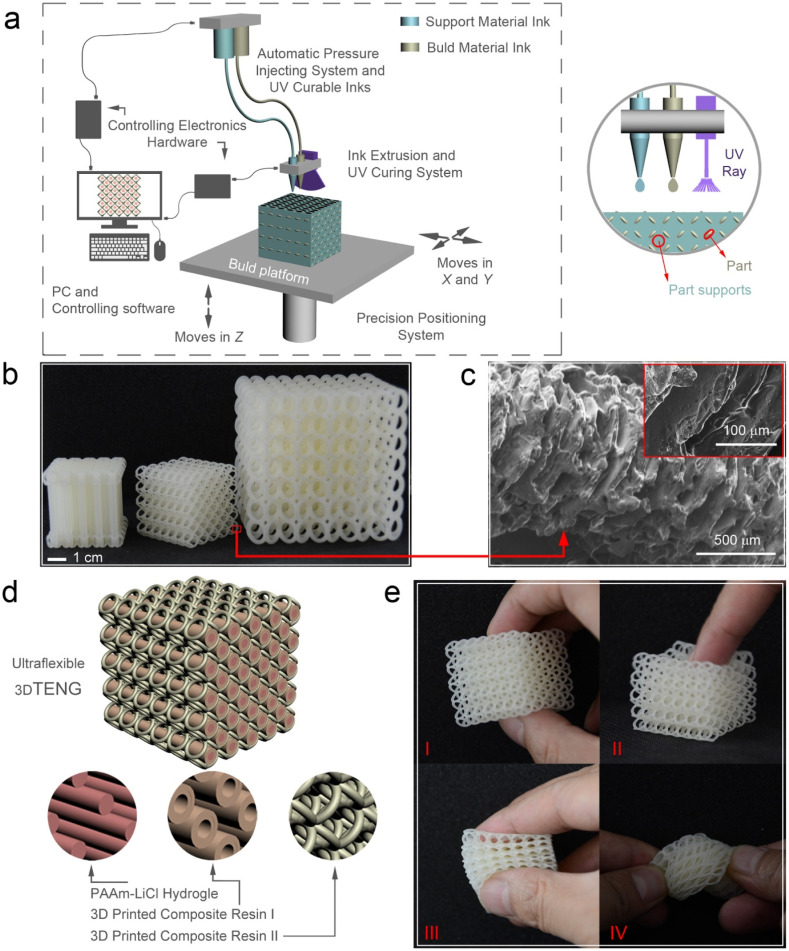
Structure and fabrication process of the ultraflexible 3D-TENG, (a) schematic illustration of the hybrid UV-assisted 3D-printing system used for fabricating ultraflexible components. (b) Digital photograph of the fabricated ultraflexible 3D-printed structures. (c) SEM images showing the layered microstructure of the printed ultraflexible parts. (d) Schematic design and assembly of the ultraflexible 3D-TENG device. (e) Mechanical flexibility and deformation tests demonstrating the ultraflexible characteristics of the printed structures. Figure reprinted with permission from ref. 177 © 2018 Elsevier Ltd. All rights reserved.

A highly adaptable three-dimensional TENG (3D-TENG) was developed to provide sustainable energy for wearable electronics, artificial intelligence systems, and internet-of-things (IoT) devices *via* biomechanical energy harvesting (Fig. 7).^177^ The device was constructed with an innovative hybrid UV-assisted 3D-printing method that combines liquid photopolymer resins and support materials in a singular additive-manufacturing process. Printed ultraflexible structures featuring internal supporting frameworks achieved excellent structural precision (<10 µm), and the subsequent removal of the support materials yielded highly bendable triboelectric components. The 3D-TENG utilized printed resin layers as triboelectric materials and ionic hydrogels as the electrification layer and electrode. Due to its optimized 3D structure and Maxwell displacement-current-driven mechanism, the device attained a peak power density of 10.98 W m^−3^ and a transferred charge density of 0.65 mC m^−3^ at a low working frequency of approximately 1.3 Hz. The system effectively energized multiple self-sustaining wearable and portable devices, such as SOS distress alarms, smart lighting shoes, temperature sensors, and smartwatches, illustrating the considerable potential of hybrid 3D-printed TENGs for multifunctional self-powered wearable systems in real-world applications.^177^

### Challenges and outlooks

The widespread adoption of wearable electronics over the last decade has driven an increasing demand for lightweight, flexible, and self-sustaining power systems. Researchers have created advanced wearable gadgets, including flexible sensors, displays, integrated circuits, batteries, supercapacitors, and energy harvesters, intended for use in healthcare, human–machine interaction, and smart textiles. Nonetheless, a significant issue remains ensuring a constant, dependable power supply necessary for uninterrupted operation. Contemporary wearable electronics, such as smartwatches and wireless earphones, necessitate hundreds to thousands of milliwatt-hours of energy; most flexible batteries and wearable energy harvesters offer relatively low energy and power outputs.

Significant advancements have been made in wearable energy-storage technology, particularly in flexible batteries and supercapacitors. Flexible supercapacitors have exhibited energy and power densities nearing those of traditional rigid devices. Notwithstanding this advancement, significant trade-offs persist between flexibility and electrochemical performance. Flexible batteries often exhibit reduced energy capacity, limited power output, and reduced mechanical durability, as the structural changes required for flexibility can undermine electrode integrity and electrochemical efficiency. Consequently, numerous commercial wearable gadgets continue to depend on cumbersome, inflexible batteries owing to their durability, established manufacturing infrastructure, and interoperability with existing electronic systems. Further investigation of other technologies, such as supercapacitors, should be considered.

Wearable energy harvesters, such as solar, thermoelectric, piezoelectric, triboelectric, electromagnetic, and bioelectrochemical systems, have garnered considerable interest. These technologies seek to transform bodily movement, thermal energy, solar radiation, and biological processes into electrical energy. Theoretical studies indicate that wearable systems may harvest significant energy from human activities and the environment; however, real devices currently have modest conversion efficiency and limited daily energy output. Most wearable harvesters produce less than 5 mW cm^−2^ and typically necessitate constant motion, substantial temperature differentials, or vigorous physical exertion to function optimally. Their sporadic energy supply and limited efficiency render them inadequate for powering most contemporary wearable gadgets independently.

Obstacles impede the commercialization of wearable energy devices. Flexible energy devices frequently face challenges related to mechanical durability, washability, long-term stability, biocompatibility, scalability, and production costs. Materials used in flexible devices may deteriorate under continuous bending, stretching, or exposure to the environment. The absence of standardized testing methodologies for flexibility, stretchability, and durability hinders equitable technology comparison and impedes industrial adoption. A significant barrier is the “technological gridlock” between academia and business, wherein promising laboratory-scale technologies face challenges in assimilating into established manufacturing and commercial processes.

To address these constraints, scientists should increasingly focus on integrated self-sustaining wearable systems that integrate energy harvesting, energy storage, and ultra-low-power electronics. Innovative solutions encompass multifunctional technologies, including self-charging supercapacitors, photo-rechargeable batteries, and self-powered sensors. Improvements in materials engineering, device architecture, power-management circuits, and additive manufacturing are expected to enhance efficiency, flexibility, and durability. Implementing uniform testing standards and enhancing collaboration between academics and industry will be crucial for converting wearable energy technologies from laboratory prototypes into viable commercial solutions.

Notwithstanding significant advancements, considerable obstacles persist, including low energy density, interfacial resistance, inadequate long-term mechanical durability, limited scalability, electrolyte instability, and ineffective energy harvesting. The advancement of multifunctional materials, highly conductive porous structures, self-healing electrolytes, scalable production techniques, and integrated energy-harvesting/storage systems is anticipated to expedite the commercialization of next-generation wearable electronics and self-sustaining flexible energy systems.

## Conclusions

Flexible supercapacitors have become one of the most promising energy-storage technologies for wearable and portable electronics due to their excellent power capability, rapid charging/discharging, long cycling life, and mechanical flexibility. Significant advances in conductive polymers, carbon nanomaterials, MXenes, metal oxides, MOFs, hybrid composites, and biodegradable starch-derived materials have greatly improved the electrochemical performance and mechanical adaptability of FSC electrodes. The incorporation of hybrid nanostructures combining EDLC and pseudocapacitive mechanisms has proven highly effective for enhancing capacitance, energy density, conductivity, and structural stability.

Recent developments in advanced fabrication technologies, including direct ink writing, inkjet printing, microfluidic spinning, wet spinning, screen printing, laser-assisted fabrication, and hybrid 3D printing, have enabled the production of highly flexible, lightweight, and architecturally controlled electrode systems. These techniques provide precise control over porosity, mass loading, ion transport pathways, and electrode geometry, which are critical for improving electrochemical performance in compact wearable devices. Furthermore, the emergence of ultra-flexible and multifunctional architectures, such as coaxial fiber-shaped supercapacitors and textile-based electrodes, demonstrates strong potential for integration into smart wearable systems.

The integration of FSCs with wearable energy harvesters, particularly TENGs, has opened new opportunities for developing self-powered and energy-autonomous electronic systems. Self-charging power systems capable of harvesting biomechanical energy and storing it directly in flexible supercapacitors represent an important step toward sustainable wearable electronics. Additionally, ionic liquid gel polymer electrolytes have shown considerable promise because of their high ionic conductivity, wide electrochemical stability windows, thermal stability, flexibility, and self-healing properties, making them highly suitable for next-generation FSCs.

Despite these advances, several challenges continue to limit practical commercialization. These include insufficient energy density compared to commercial batteries, interlayer resistance in thick printed electrodes, restacking effects in layered nanomaterials such as MXenes, poor long-term mechanical durability, limited washability, electrolyte leakage, and low energy-harvesting efficiency. In addition, scalable manufacturing, cost-effective processing, standardized testing protocols, and compatibility with existing industrial infrastructure remain critical issues.

Future research should focus on the rational design of multifunctional hybrid materials, hierarchical porous architectures, self-healing and biodegradable electrolytes, and integrated energy harvesting-storage systems with improved mechanical robustness and electrochemical efficiency. The development of scalable additive-manufacturing technologies and low-cost, sustainable materials will also be essential for enabling commercial wearable energy-storage systems. Through continued interdisciplinary efforts in materials science, electrochemistry, manufacturing, and device engineering, flexible supercapacitors and self-powered wearable systems are expected to play a central role in the next generation of intelligent, portable, and sustainable electronics.

## Conflicts of interest

The authors declare no competing interests.

## Data Availability

There are no data generated in this review. All data are available in the text.
